# Editorial: Health, wellbeing, performance and learning in extreme contexts and natural environments

**DOI:** 10.3389/fpsyg.2025.1724555

**Published:** 2025-10-29

**Authors:** Royce L. Willis, Vinathe Sharma-Brymer, Matthew Leach, Eric Brymer

**Affiliations:** ^1^Faculty of Health, Southern Cross University, Coffs Harbour, NSW, Australia; ^2^School of Law and Society, University of the Sunshine Coast, Maroochydore, QLD, Australia; ^3^Health and Community Services, TAFE SA, Noarlunga Centre, SA, Australia; ^4^Manna Institute, New England University, Lismore, NSW, Australia

**Keywords:** Nature-based physical activity, Adventure psychology, mindfulness, Risk Perception, affordances, wellbeing, human-nature relationships, extreme environments

Human life is increasingly organized around built, controlled, and technologically mediated environments. Yet formative experiences often arise in nature and contexts that can be extreme, uncertain, or challenging. This Research Topic examines how such environments shape human health, wellbeing, performance, and learning. The seven contributions to Research Topic highlight mechanisms of effect, reconceptualization of adventure and risk, the role of place and relationality, and calls to reintegrate nature into personal and societal life.

The first thread concerns mechanisms of psychological change. Chouja et al. present a scoping review using an Ecological Dynamics lens to link nature-based physical activity, mindfulness, and wellbeing. The review suggests that nature-based physical activities can facilitate mindfulness associated with wellbeing outcomes, while calling for stronger causal designs. Complementing this, Gürer et al. compared adolescent rock climbers with matched peers and found lower Separation Anxiety Disorder and Generalized Anxiety Disorder scores among climbers. Extending this thread, Brymer et al. argue that adventure should be redefined through an ecological lens: not merely risk plus challenge, but a relational, multidimensional process affording opportunities for growth. Adding a broader evidence base on participation drivers, Hornby et al. synthesize 35 studies and reveal that participation is shaped by how individuals perceive and negotiate risk, the psychological needs extreme sports fulfill, and the meaningful experiences people derive from engaging with nature in high-stakes contexts.

The second thread centers on place, relationality, and affordances. From a Perspective piece, Sharma-Brymer et al. argue that socio-emotional wellbeing in Forest School may be strengthened when Western notions of affordances are integrated with Indigenous place-based relationality. Natural environments and landscapes are treated not as neutral backdrops but as active participants in children's development. This emphasis on place resonates with the ecological reconceptualization of adventure, as both highlight how meaning is co-constructed through reciprocal relationships between people and their environments.

The Research Topic also includes work that situates nature within broader societal and philosophical debates. Willis et al. argue for reintegrating nature into everyday life and emphasize that ecological and human flourishing are inseparable. The authors urge researchers and practitioners to move beyond instrumental framings of nature as “treatment” and instead build cultural, educational, and policy contexts that embed reciprocal human–nature relationships, linking individual wellbeing to the planetary systems that sustain it.

Finally, studies of risk perception and decision-making show that extreme contexts are dynamic environments that demand agency. In a pilot with experienced mountain bikers, Chilton and Robinovitch report that willingness to ride raised ramps is best explained by the product of perceived probability of falling and perceived probability of injury if a fall occurs; notably, riders generally declined when this perceived injury risk exceeded ~13%. This work highlights that psychological outcomes in adventure contexts cannot be separated from choices made under environmental constraints.

Collectively, the contributions caution against a simplistic “nature exposure equals benefit” logic. This body of work shows that the quality of engagement—mindful attention, immersion, relational attunement, risk negotiation—often matters as much as duration or frequency. While “dose–response” framings are common, they are most useful when quality is recognized as part of the dose. It is not merely the hours outdoors that matter, but whether environments invite attentiveness, provide challenge, or enable reciprocity.

These collective works also provide some valuable converging insights. First, mindfulness and attention regulation recur as mechanisms, whether implicitly fostered during physical activity or explicitly theorized as mediators of wellbeing. Second, risk and decision-making are integral to benefits in extreme contexts, particularly during adolescence, when negotiating boundaries supports development. Third, place and cultural context shape outcomes; Forest School perspectives underscore that socio-emotional wellbeing cannot be divorced from histories and unique identities of land and relational worldviews. Finally, broader integrative perspectives remind us that the health of humans and ecosystems are entwined.

The implications are practical. Designers of outdoor and adventure programs should prioritize quality of engagement over exposure time, deliberately cultivating immersion, attentional depth, relational engagement, and participant agency. Beyond this, designers should calibrate challenge and safety through shared decision-making and participant-led risk-calibration tools that support perceptual learning. Educators should embed place-based, culturally responsive activities co-designed with Indigenous partners. Policymakers should expand equitable access to high-quality natural affordances, especially in cities, and fund programs that scaffold meaningful contact. To enable replication and scaling, it is imperative that stakeholders report how “quality × time” is operationalized (e.g., immersion indices, affordance mapping), together with governance arrangements and contextual details.

Future research should strengthen causal inference by employing longitudinal and intervention designs that vary quality × time. New measures are needed to capture aspects of “dose” quality, including immersion, attentional depth, and relational richness. A greater diversity of populations and contexts is critical, including urban settings, non-Western cultural contexts, and groups that are underrepresented in nature and adventure research. Mixed methods designs that combine physiological data, qualitative accounts, and ecological assessment would also yield a more holistic understanding of the field. Ethical and relational dimensions also require attention, including how ecological identity, cultural background, and place histories mediate human experiences.

This Research Topic shows a field expanding across psychology, sport, education, and environmental studies. Rather than converging on a single model, the contributions map a diverse terrain—from empirical analyses of risk perception to ecological reconceptualization's of adventure, to culturally sensitive accounts of place, to calls for nature reintegration. Coherence lies in recognizing extreme contexts and natural environments as powerful teachers that shape who we are, how we perform, and how we connect with each other and the more-than-human world.

This Research Topic will hopefully encourage researchers to deepen work on mechanisms, broaden contexts and populations, and integrate quality of experience into future designs. More broadly, we invite critical reflection on the reciprocal relationship between human flourishing and ecological wellbeing, and on designing environments, policies, and practices that honor both.

